# External Validation of Deeplasia for Automated Bone Age Assessment Compared with Four Commercial AI Systems

**DOI:** 10.3390/diagnostics16162568

**Published:** 2026-08-14

**Authors:** Johanna Pape, Roland Pfäffle, Franz Wolfgang Hirsch, Maciej Rosolowski, Daniel Gräfe

**Affiliations:** 1 Department of Pediatric Radiology, University Hospital Leipzig, 04103 Leipzig, Germany; franzwolfgang.hirsch@medizin.uni-leipzig.de (F.W.H.); daniel.graefe@medizin.uni-leipzig.de (D.G.); 2 Department of Diagnostic and Interventional Radiology, University Hospital Leipzig, 04103 Leipzig, Germany; 3 Department of Pediatrics, University Hospital Leipzig, 04103 Leipzig, Germany; roland.pfaeffle@medizin.uni-leipzig.de; 4 Institute for Medical Informatics, Statistics and Epidemiology, Leipzig University, 04103 Leipzig, Germany; maciej.rosolowski@imise.uni-leipzig.de

**Keywords:** bone age, artificial intelligence, softwares, pediatrics, radiology

## Abstract

**Background/Objectives**: Artificial intelligence (AI)-based systems enable automated bone age (BA) assessment according to the Greulich and Pyle (G&P) method with expert-level performance. Deeplasia is a recently introduced deep learning-based approach that demonstrated promising results in previous studies. This study aimed to externally validate Deeplasia for G&P-based BA and chronological age (CA) estimation. **Methods**: This retrospective single-center study included two independent cohorts. For BA assessment, 306 children and adolescents aged 1–18 years were analyzed using the mean rating of three expert readers as the reference standard. For CA assessment, 1653 children and adolescents undergoing hand radiography after trauma were included after exclusion of pathological findings. Deeplasia was compared with four CE-certified AI systems. Performance was evaluated using mean error, mean absolute error (MAE), root mean squared error (RMSE), and Bland–Altman limits of agreement. **Results**: Deeplasia achieved a very good overall agreement with the human reference standard, with the lowest RMSE (0.59 years in boys, 0.55 years in girls) and MAE (0.45 years in boys, 0.43 years in girls). However, no significant differences between the AI systems were observed within the age range representing 90% of the clinically relevant cohort. Estimation of the CA was substantially less accurate than G&P-based BA assessment across all systems. All programs showed systematic overestimation of CA, particularly in adolescent girls. **Conclusions**: Compared to commercial AI systems, Deeplasia demonstrated excellent external validity for automated G&P-based BA assessment. However, the findings again highlight the intrinsic limitations of G&P-based models for precise CA estimation in contemporary pediatric populations.

## 1. Introduction

Artificial intelligence (AI) has enabled automated, reproducible, and substantially faster bone age (BA) assessment according to the Greulich and Pyle (G&P) method, while maintaining expert-level performance. A major impetus for the development of AI-based methods in this field was the Radiological Society of North America (RSNA) 2018 challenge [1]. As a result, numerous algorithms have been developed, several of which are currently approved CE (Conformité Européenne) medical devices for clinical use on the European market [2].

Since the RSNA challenge, both AI architectures and their performance have continued to evolve, with new systems still being published. A recently introduced program, Deeplasia, demonstrated superior performance compared with established commercial market leaders and has already been validated in a clinically relevant and diagnostically challenging population: children with bone dysplasia [3]. In addition, recent studies have evaluated Deeplasia in broader pediatric cohorts with growth disorders, as well as in independent international populations, further supporting the robustness of the approach across different clinical contexts [4,5]. In this cohort, the software achieved promising overall results.

However, external validation in an independent cohort of children with non-dysplastic hands is still lacking for a comprehensive assessment of the clinical value of Deeplasia. Furthermore, there is limited evidence on the extent to which an algorithm based on the G&P reference standard—a historical cohort of children from the first half of the 20th century—accurately reflects the chronological age (CA) of healthy contemporary Central European children [6]. Against the backdrop of known secular trends in growth and skeletal maturation, this remains a significant unresolved issue.

The aim of the present study was therefore to externally validate Deeplasia for the prediction of both G&P BA and CA in a contemporary pediatric cohort without bone dysplasia. For the comparative analysis, we hypothesized that the accuracy of Deeplasia would not differ significantly from that of the established commercial AI systems.

## 2. Materials and Methods

### 2.1. Study Design

This retrospective study included two independent cohorts for BA and CA assessment. Ethical approval was obtained by the local ethics committee (EK 46/20).

### 2.2. Bone Age Cohort

For the comparative assessment of G&P bone age, a previously established and published validation cohort was used [7]. In the original cohort, stratified random sampling was performed from 5612 available hand radiographs according to chronological age and sex, with nine patients randomly selected per sex and one-year age stratum. This resulted in a predefined sample of 306 children and adolescents (153 female and 153 male patients). Because the present study represents a secondary analysis of this fixed cohort, no separate a priori sample-size calculation was performed for the current analysis. The human G&P reference standard was defined as the mean of the G&P assessments independently performed by three expert readers.

### 2.3. Chronological Age Cohort

For the comparative estimation of CA, a cohort previously described elsewhere was used [8]. The cohort comprised 1653 children and adolescents aged 1–18 years who underwent radiography of the left or right hand to rule out trauma-related sequelae. All radiographs were screened by a physician, and cases with pathological findings (fractures, dislocations, or dysmorphisms) were excluded. In this cohort, CA served as the reference standard.

### 2.4. Radiographs

Image acquisition was performed exclusively at a single pediatric radiology center (Leipzig University Hospital, a tertiary referral center) using an Axiom Aristos FX system (Siemens Healthineers, Erlangen, Germany). Radiographs were obtained without an anti-scatter grid and with a 0.1-mm copper filter. By default, the left hand, including the wrist, was imaged in a posteroanterior projection; if unavailable, the right hand was used as a substitute.

### 2.5. Artificial Intelligence Systems

In addition to the non-CE-certified software Deeplasia v1.0 Institut for Genomic Statistics and Bioinformatics, Bonn, Germany), four CE-certified software platforms were employed: BoneXpert v3.2.2 (Visiana, Hørsholm, Denmark), PANDA v1.13.21 (IBLabs, Vienna, Austria), BoneView v2.3.1.1 (Gleamer, Paris, France), and TechCare Kids (Milvue, Paris, France). Apart from BoneView, all AI applications were integrated via a local stand-alone service, whereas BoneView analyses were performed using a cloud-based solution. BoneView does not evaluate hand radiographs for CA below 3 years or BA above 17 years. The intended use of PANDA includes children aged ≥3 years and <16 years (girls) or <17 years (boys).

Deeplasia consists of an ensemble of three EfficientNet-based models, with the largest individual model containing approximately 23 million parameters—substantially fewer than earlier end-to-end models with over 80 million parameters cited in the original Deeplasia study. A complete bone-age assessment with Deeplasia, including image preprocessing and prediction by the three-model ensemble, can be performed in less than 10 s per radiograph on consumer-grade hardware using CPU computation alone, such as a standard laptop, without GPU acceleration.

### 2.6. Statistical Analysis

Statistical analyses were performed using RStudio version 2025.09.2 (Posit Software, PBC, Boston, Massachusetts, USA). Continuous variables are reported as mean ± standard deviation (SD) or median (interquartile range), as appropriate. All age values are reported in years.

To quantify deviations between AI-based estimates and the reference BA, the mean error (ME), mean absolute error (MAE), and root mean squared error (RMSE) were calculated. In addition, limits of agreement (LoA) according to Bland–Altman were determined as ±2 SD of the differences and reported as the total range (4 × SD). Missing values were handled using listwise deletion.

Methodological agreement between AI systems and the reference BA was further assessed using Bland–Altman analyses based on linear mixed-effects models implemented in the MethComp package version 1.30.0. Analyses were conducted separately for male and female patients. Constant difference models were applied, and the differences were plotted against the mean of the AI-based estimate and the reference BA.

For the analysis of CA estimation, analogous error metrics (ME, MAE, RMSE, and LoA) were calculated. Age-dependent deviations between estimated BA and CA were visualized using sliding age windows (±1.5 years). For the comparative analysis, the null hypothesis (H_0_) was that there was no systematic difference in absolute prediction error between Deeplasia and the respective comparator AI system. Differences in absolute prediction error between Deeplasia and each commercial comparator were assessed using two-sided paired Wilcoxon signed-rank tests with Holm correction across the four comparisons. Post hoc power was estimated using 10,000 nonparametric patient-level bootstrap replicates, with the four tests and Holm adjustment repeated in each replicate. Analyses were performed (a) in the entire dataset, where the number of included cases varied according to image rejection by the respective AI systems, and (b) in a subgroup covering approximately 90% of all bone age examinations (4.8–15.5 years for females and 4.9–17.0 years for males), in which every case was evaluated by all AI systems [9].

All statistical tests were two-sided, and a *p*-value < 0.05 was considered statistically significant.

## 3. Results

### 3.1. Bone Age Assessment

Within the established cohort, 306 patients aged 1–18 years were included, with an equal distribution of male and female participants [7] (Supplementary Table S1). BoneView rejected 62 radiographs, whereas Deeplasia and PANDA analyzed all images. Deeplasia demonstrated the lowest RMSE (0.59 years in boys and 0.55 years in girls), as well as the lowest MAE (0.54 years in boys and 0.43 years in girls), as shown in Table 1.

**Figure 1:**
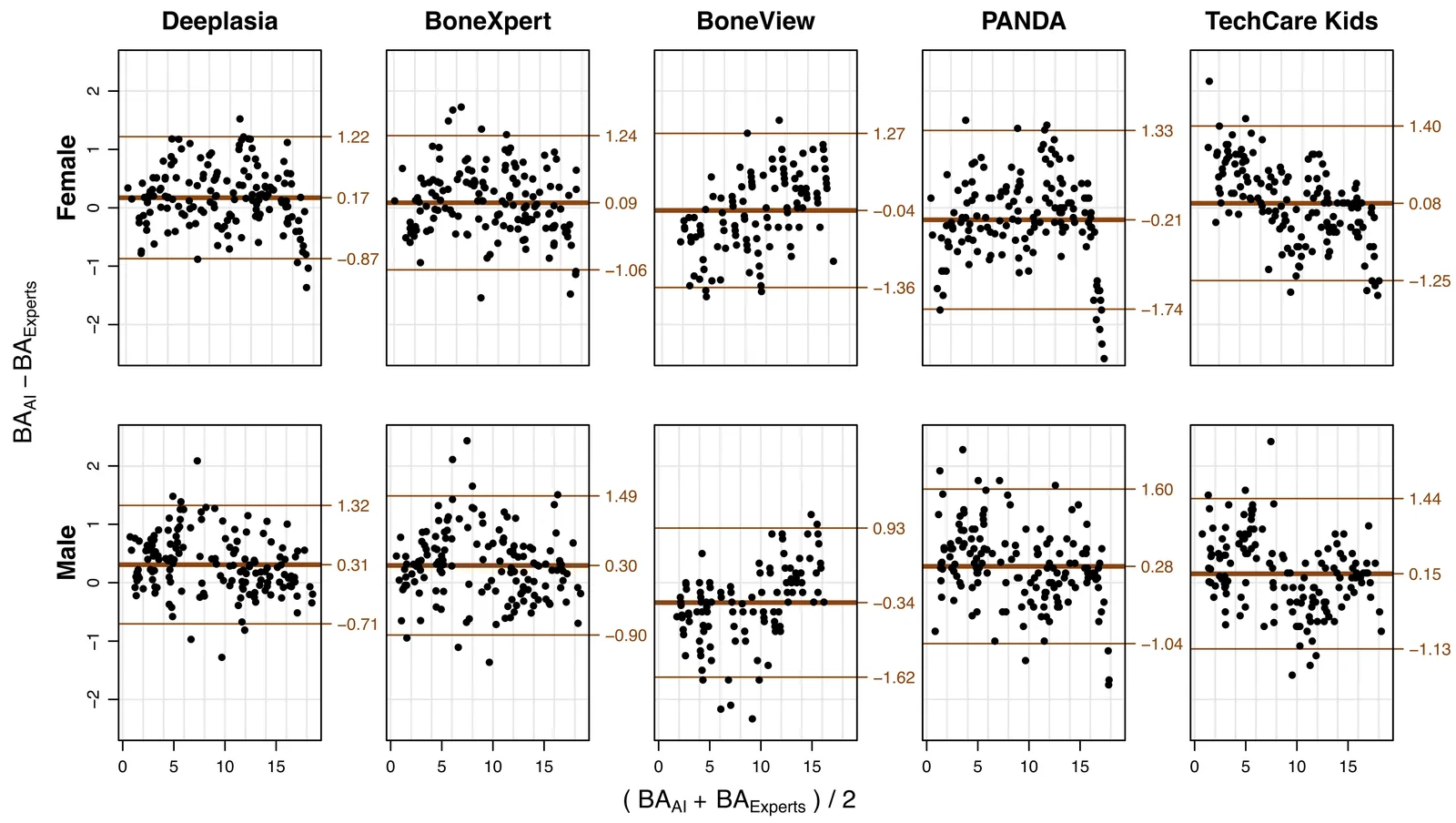
Bland–Altman plots of five artificial intelligence (AI) programs for Greulich and Pyle-based bone age (BA) assessment compared with the mean estimates of three expert readers in an age- and sex-stratified patient cohort. The central and outer lines indicate the mean difference and limits of agreement, respectively.

The variance of BA estimates remained largely constant across childhood and adolescence. However, Deeplasia—like TechCare Kids and PANDA—tended to underestimate BA in older adolescent girls compared with the human reference readers. In Bland–Altman analyses, Deeplasia exhibited the narrowest LoA, with total ranges of 2.09 years for girls and 2.03 years for boys, as shown in Figure 1. Detailed subgroup performance data can be found in Supplementary Table S2.

Regarding the MAE, after adjustment for multiple comparisons, only the lower MAE of Deeplasia compared with PANDA and TechCare Kids reached statistical significance (*p* < 0.001 and *p* = 0.003, respectively; all other comparisons: *p* ≥ 0.4). In the subgroup comprising the most commonly encountered age range, the MAE did not differ significantly between vendors (*p* = 1; Table 2).

In the complete cohort, the Holm-adjusted bootstrap post hoc power was 95.2% for the comparison of Deeplasia with PANDA and 84.0% for Deeplasia versus TechCare Kids. For the comparisons with BoneView and BoneXpert, power was lower at 40.8% and 38.8%, respectively.

### 3.2. Chronological Age Assessment

Within the established cohort, 1653 patients aged 1–18 years were included, of whom 52% were boys (Supplementary Table S1). Compared with G&P-based BA estimation, the inaccuracy of CA estimation was nearly twice as high (RMSE: 1.22 vs. 0.64 years; MAE: 0.92 vs. 0.50 years; LoA range: 2.78 vs. 2.41 years).

**Figure 2:**
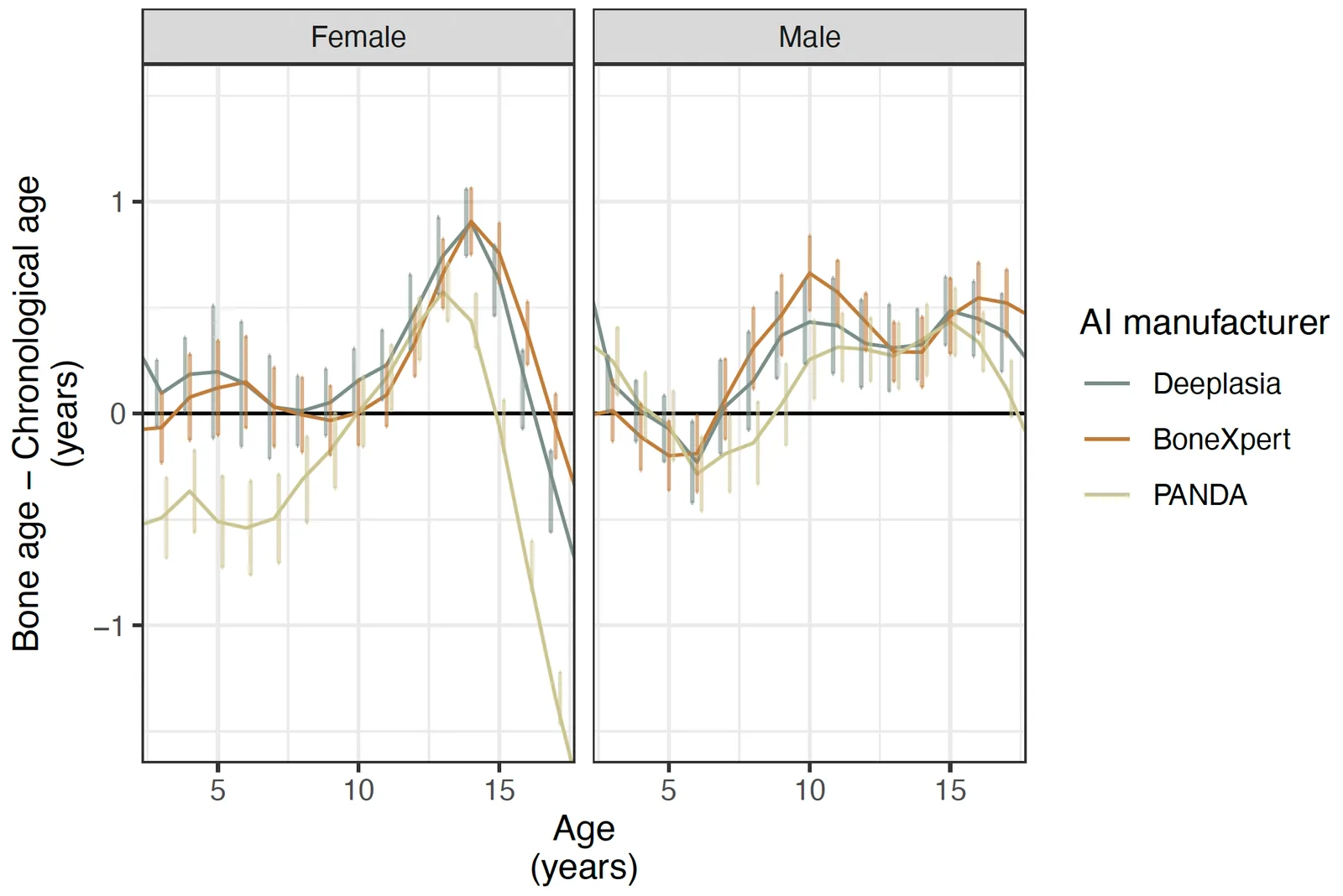
Age- and sex- dependent differences between artificial intelligence (AI)-estimated chronological age fand chronological age in the contemporary cohort of children and adolescents. Positive values indicate overestimation and negative values indicate underestimation of chronological age. Please note that the intended use of PANDA includes only children aged ≥3 years and <16 years (girls) or <17 years (boys). Curves show mean differences calculated using sliding age windows of ±1.5 years.

All three programs showed a pronounced overestimation of CA in adolescent girls, as well as a moderate overestimation in boys throughout childhood and adolescence, amounting to approximately 11 months and 5 months, respectively (Figure 2). Only PANDA demonstrated an overestimation of CA in young girls.

## 4. Discussion

The present study validates the performance of Deeplasia in a healthy, contemporary pediatric cohort and compares it with four CE-certified AI-based systems for automated BA assessment.

Deeplasia demonstrated consistently high agreement with the human reference standard and showed equal or superior performance across all major error metrics, with particularly narrow LoA. The age- and sex-stratified subgroup analysis broadly corroborated the overall findings presented in Table 1, with Deeplasia showing consistently low error metrics and comparatively narrow limits of agreement, particularly in girls younger than 6 years and in adolescents older than 12 years. However, performance differences were not uniform across age groups, as Deeplasia performed similarly to several comparator systems in children aged 6–12 years and individual competitors achieved slightly lower errors in selected sex-specific strata, demonstrating that its overall advantage did not translate into uniform superiority in every subgroup.

A plausible explanation repeatedly proposed in the literature for the higher variability of bone age in male patients also observed in our study is the longer and more variable course of skeletal maturation in boys [10] and greater interindividual variability in pubertal timing [11].

For the entire cohort, the MAE of Deeplasia was significantly lower than that of TechCare Kids and PANDA. However, concerning BoneView, this finding should be interpreted with caution, as BoneView, unlike the other four AI systems, did not accept children younger than 3 years for analysis. Consequently, the number and composition of analyzed cases differed between vendors, which methodologically limits the direct comparability of performance metrics across the entire cohort. Accordingly, when restricting the analysis to the age range representing approximately 90% of examinations encountered in clinical practice, no significant differences in MAE were observed between the five AI systems.

However, the absence of statistically significant differences should not be interpreted as evidence of equivalence between the systems. The Holm-adjusted post hoc power for the 4 pairwise comparisons ranged between 34% and 88% (0.7–8.3%), reflecting the very small observed between-system differences and indicating that small differences may have remained undetected. Accordingly, a Type II error cannot be excluded. At the same time, the observed mean pairwise differences in absolute error were small (≤0.036 years), suggesting that any undetected differences are likely to be quantitatively limited within this clinically relevant age range.

In the context of AI-based BA assessment, such independent external validation is essential because performance estimates derived from internal or vendor-curated datasets may not generalize to routine clinical populations and may be affected by spectrum bias or overfitting [12]. Recent validation studies of commercial and open-source BA tools have likewise emphasized that clinically meaningful evaluation requires testing in independent cohorts that reflect real-world case mix rather than optimized benchmark datasets [3,4,13,14]. Systematic over- or underestimation may affect the clinical classification of skeletal maturation and subsequent treatment or follow-up decisions, particularly at the extremes of the pediatric age range or near relevant decision thresholds. AI-derived estimates should therefore be interpreted in the clinical context and reviewed by an experienced reader when discordant with the overall clinical presentation.

Because the present study used an independent cohort with age- and sex-stratified sampling, it directly addresses a central methodological weakness of many AI validation studies, namely limited external generalizability. The observed performance of Deeplasia is therefore more likely to reflect routine clinical conditions than highly selected development environments [3].

Across all software evaluated in this study, a systematic deviation between BA estimates and true CA was observed in healthy children. This age- and sex-dependent effect was most pronounced in cent girls, with mean deviations of up to approximately 1 year. Because this pattern was observed across all vendors, it is unlikely to represent a software-specific defect; rather, it more plausibly reflects an inherent limitation of using the G&P atlas as the underlying reference framework. The G&P atlas was derived from white North American children of comparatively favorable socioeconomic background examined in the 1930s to early 1940s, and its transportability to contemporary populations has long been debated. Meta-analytic and population-based studies have shown that deviations between skeletal age and CA vary by sex, ethnicity, and age group, even when the atlas is applied carefully [15,16]. The G&P method is also affected by age-dependent uncertainty and reader variability, particularly during the pubertal growth period. Little et al. reported limitations of skeletal-age-based prediction for the timing of epiphysiodesis, while Cundy et al. found inter-reader differences exceeding 2 years in 10% of children with leg-length discrepancy [17,18]. Thus, G&P bone age should not be interpreted as an error-free basis for individual treatment decisions or as a precise surrogate for chronological age.

This interpretation is also supported by the literature on secular changes in growth and pubertal timing. In particular, the timing of pubertal onset in girls has shifted earlier over recent decades, with a large systematic review and meta-analysis reporting a decrease in age at thelarche of nearly 3 months per decade from 1977 to 2013 [19]. Such secular shifts can decouple contemporary biological maturation patterns from historical atlas standards and may help explain why BA, even when estimated very accurately according to G&P criteria, does not necessarily coincide with true CA in modern populations [8]. Accordingly, AI-derived BA should be interpreted primarily as a marker of skeletal maturation within the G&P framework rather than as a direct surrogate for chronological age.

The substantially higher error observed for CA estimation than for G&P-based BA prediction further supports this distinction. AI systems may reproduce expert-level G&P assessments with high fidelity, but their ability to infer CA is fundamentally constrained by the historical and population-specific nature of the reference standard. This distinction is especially relevant in non-clinical applications such as medicolegal age estimation, where even modest systematic error may have important consequences [20]. Therefore caution by forensic and medicolegal studies is recommended when G&P-based hand radiograph assessment is used to estimate CA in living individuals, particularly in populations that differ from the original reference sample (i.e., non-Caucasian) or in contexts requiring high individual-level accuracy [21]. Similar population-related effects have also been reported in recent evaluations of Deeplasia in geographically distinct cohorts, including children from Georgia, highlighting the importance of validating AI-based bone age systems across diverse populations [4].

From a clinical perspective, however, the error margins achieved by Deeplasia for BA determination according to G&P appear to be within the range of known interobserver variability for experienced human readers [22]. Classical studies of manual G&P reading demonstrated relevant reader dependence, influenced in part by experience and by knowledge of CA; automated systems therefore offer an important advantage by standardizing the application of the atlas and reducing reader-dependent variation [22]. This potential benefit has been confirmed in both classical validation studies of automated BA software and also in AI-based reader studies, which showed improved reproducibility and reduced inter- and intra-observer variability [22,23,24,25]. Against this background, the high fidelity of Deeplasia across the pediatric age range supports its role as a reliable decision-support tool for standardized BA assessment in clinical practice. Looking ahead, the field should move beyond the outdated G&P reference standard toward a data-driven, AI-based BA standard trained on contemporary, multiethnic pediatric populations.

Several limitations merit consideration. First, the single-center design represents both a methodological strength and a limitation. Uniform image acquisition and the use of a common expert reference standard reduced technical variability and facilitated a controlled head-to-head comparison of the evaluated AI systems. At the same time, the technical and demographic homogeneity of the cohort limits the generalizability of our findings to other institutions, imaging devices, acquisition protocols, and patient populations. Second, the CA cohort was derived from a trauma population and can therefore only be assumed to represent predominantly healthy children. Although subtle growth abnormalities cannot be entirely excluded, their effect on the cohort-level estimates may be limited if delayed and accelerated maturation are distributed without systematic selection. Third, ethnicity and socioeconomic background were not systematically recorded in this retrospective cohort and could therefore not be evaluated in subgroup analyses. This is relevant because the relationship between G&P-based BA and CA may vary across geographically, ethnically, and socioeconomically diverse populations [8,15]. The present study should therefore be regarded as an independent single-center external validation rather than evidence of universal transportability. Prospective multicenter studies involving diverse populations, imaging systems, and acquisition settings are required to assess the broader generalizability of these findings. Lastly, Deeplasia is currently not certified as a medical device in either Europe or the United States.

Deeplasia demonstrated high agreement with expert G&P-based bone-age assessments. However, the additional chronological-age analysis indicates that G&P-based skeletal-age estimates should not be interpreted as precise surrogates for chronological age in contemporary pediatric populations.

## 5. Conclusions

Deeplasia demonstrates excellent external validity for automated BA assessment. However, all G&P-based models show inherent limitations for accurate CA estimation in modern populations.

## Figures and Tables

**Table 1:** Performance of all five artificial intelligence (AI)-based systems for Greulich & Pyle-based bone age estimation compared with the human reference standard in an age- and sex-stratified pediatric cohort. Error metrics are reported in years, separately for boys and girls. Positive and negative ME values indicate overestimation and underestimation relative to the mean assessment of three expert readers, respectively.

	*n*	RMSE		MAE		ME		LoA	
		M	F	M	F	M	F	M	F
BoneView	244	0.72	0.66	0.54	0.55	−0.34	−0.04	2.55	2.64
BoneXpert	299	0.66	0.58	0.51	0.46	0.30	0.09	2.38	2.30
Deeplasia	306	0.59	0.55	0.45	0.43	0.31	0.17	2.03	2.09
PANDA	306	0.72	0.79	0.54	0.58	0.28	−0.21	2.65	3.06
TechCare Kids	305	0.66	0.66	0.52	0.53	0.15	0.08	2.57	2.65

Abbreviations: M male; F female; RMSE root mean squared error; MAE mean absolute error; ME mean error; LoA limits of agreement (span).

**Table 2:** Performance of all five artificial intelligence (AI)-based systems for Greulich & Pyle-based bone age estimation compared with the human reference standard in an age- and sex-stratified pediatric cohort. Only the age groups most commonly encountered in clinical practice (90% interval) were included. Error metrics are reported in years, separately for boys and girls.

	*n*	RMSE		MAE		ME		LoA	
		M	F	M	F	M	F	M	F
BoneView	206	0.73	0.63	0.53	0.52	−0.30	−0.01	2.67	2.53
BoneXpert	206	0.71	0.60	0.56	0.47	0.34	0.18	2.53	2.31
Deeplasia	206	0.64	0.58	0.49	0.47	0.31	0.26	2.24	2.09
PANDA	206	0.68	0.62	0.52	0.48	0.24	0.00	2.54	2.47
TechCare Kids	206	0.69	0.58	0.54	0.47	0.10	−0.01	2.74	2.35

Abbreviations: M male; F female; RMSE root mean squared error; MAE mean absolute error; ME mean error; LoA limits of agreement (span).

## Data Availability

Data are available upon reasonable request from the corresponding author.

## Supplementary Materials

The following supporting information can be downloaded at: https://www.mdpi.com/article/10.3390/diagnostics16162568/s1, Supplementary Table S1. Epidemiological characteristics of the study cohorts. Supplementary Table S2. Age- and sex-stratified performance metrics of the evaluated bone age estimation software programs.

## Abbreviations

The following abbreviations are used in this manuscript:

AI	Artificial Intelligence
BA	Bone Age
CA	Chronological Age
G&P	Greulich und Pyle
CE	Conformité Européenne
F	Female
LoA	Limits of Agreement
M	Male
MAE	Mean Absolute Error
ME	Mean Error
SD	Standard Deviation
