# Supplementary material for: External Validation of Deeplasia for Automated Bone Age Assessment Compared with Four Commercial AI Systems
Source: Diagnostics (Basel). 2026 Aug 14;16(16):2568. doi: 10.3390/diagnostics16162568 (PMC13512856; doi:10.3390/diagnostics16162568)
Supplement: Supplementary file 1 [file diagnostics-16-02568-s001.zip › Supplemental Table S2.pdf]

Supplemental Table S2. Age- and sex-stratified performance metrics of the evaluated bone age estimation software programs.

Vendor	*Age group*	*Gender*	*RootMeanSquaredError*	*MeanAbsoluteError*	*MeanError*	*LoA_range*
BoneView	Below 6	Female	0,76	0,63	-0,61	1,89
BoneView	Below 6	Male	0,71	0,59	-0,59	1,60
BoneXpert	Below 6	Female	0,59	0,45	0,12	2,33
BoneXpert	Below 6	Male	0,50	0,41	0,26	1,72
Deeplasia	Below 6	Female	0,46	0,36	0,15	1,76
Deeplasia	Below 6	Male	0,55	0,45	0,38	1,62
PANDA	Below 6	Female	0,69	0,56	-0,33	2,46
PANDA	Below 6	Male	0,83	0,65	0,55	2,49
TechCare Kids	Below 6	Female	0,81	0,68	0,63	2,08
TechCare Kids	Below 6	Male	0,68	0,53	0,41	2,16
BoneView	6 to 12	Female	0,65	0,54	-0,01	2,62
BoneView	6 to 12	Male	0,86	0,64	-0,55	2,67
BoneXpert	6 to 12	Female	0,61	0,51	0,25	2,25
BoneXpert	6 to 12	Male	0,90	0,75	0,52	3,00
Deeplasia	6 to 12	Female	0,63	0,52	0,34	2,15
Deeplasia	6 to 12	Male	0,75	0,60	0,44	2,46
PANDA	6 to 12	Female	0,66	0,55	0,14	2,61
PANDA	6 to 12	Male	0,73	0,58	0,31	2,68
TechCare Kids	6 to 12	Female	0,61	0,49	0,03	2,45
TechCare Kids	6 to 12	Male	0,76	0,61	0,17	3,01
BoneView	Above 12	Female	0,59	0,50	0,29	2,09
BoneView	Above 12	Male	0,48	0,36	0,12	1,87
BoneXpert	Above 12	Female	0,54	0,42	-0,11	2,13
BoneXpert	Above 12	Male	0,47	0,36	0,10	1,83
Deeplasia	Above 12	Female	0,53	0,41	0,03	2,13
Deeplasia	Above 12	Male	0,41	0,31	0,12	1,61
PANDA	Above 12	Female	0,97	0,64	-0,45	3,46
PANDA	Above 12	Male	0,59	0,42	0,03	2,39
TechCare Kids	Above 12	Female	0,58	0,44	-0,32	1,94
TechCare Kids	Above 12	Male	0,52	0,41	-0,08	2,07
