# Supplementary material for: External Validation of Deeplasia for Automated Bone Age Assessment Compared with Four Commercial AI Systems
Source: Diagnostics (Basel). 2026 Aug 14;16(16):2568. doi: 10.3390/diagnostics16162568 (PMC13512856; doi:10.3390/diagnostics16162568)
Supplement: Supplementary file 1 [file diagnostics-16-02568-s001.zip › Supplemental Table S1.pdf]

Supplemental Table S1. Epidemiological characteristics of the two cohorts.
Age- and sex-stratified cohorts used to evaluate the age-estimation performance of the software programs, with Greulich and Pyle (G&P) bone age as the reference standard for the G&P cohort and chronological age as the reference standard for the Healthy cohort.

	G&P cohort (n=306)		Healthy cohort (n=1653)	
	Male	Female	Male	Female
Patients (#)	153	153	855	798
Gender-Ratio	1	1	1.1	1
Chronological Age (y)	9.4 (5.2 – 13.5)	9.6 (5.2 – 13.5)	11.8 (8.1 – 14.8)	11.6 (8.8 – 14.7)
Reference standard	G&P age, as assessed by three expert readers		Chronological age of the patients	
